# Interbasin Water Transfer, Riverine Connectivity, and Spatial Controls on Fish Biodiversity

**DOI:** 10.1371/journal.pone.0034170

**Published:** 2012-03-28

**Authors:** Evan H. Campbell Grant, Heather J. Lynch, Rachata Muneepeerakul, Muthukumarasamy Arunachalam, Ignacio Rodríguez-Iturbe, William F. Fagan

**Affiliations:** 1 United States Geological Survey (USGS) Patuxent Wildlife Research Center, Laurel, Maryland, United States of America; 2 Ecology and Evolution Department, Stony Brook University, Stony Brook, New York, United States of America; 3 School of Sustainability and Mathematical, Computational, and Modeling Sciences Center, Arizona State University, Tempe, Arizona, United States of America; 4 Sri Paramakalyani Centre for Environmental Sciences, Manonmaniam Sundaranar University, Tamilnadu, India; 5 Department of Civil and Environmental Engineering, E-Quad, Princeton University, Princeton, New Jersey, United States of America; 6 Department of Biology, University of Maryland, College Park, Maryland, United States of America; Smithsonian's National Zoological Park, United States of America

## Abstract

**Background:**

Large-scale inter-basin water transfer (IBWT) projects are commonly proposed as solutions to water distribution and supply problems. These problems are likely to intensify under future population growth and climate change scenarios. Scarce data on the distribution of freshwater fishes frequently limits the ability to assess the potential implications of an IBWT project on freshwater fish communities. Because connectivity in habitat networks is expected to be critical to species' biogeography, consideration of changes in the relative isolation of riverine networks may provide a strategy for controlling impacts of IBWTs on freshwater fish communities.

**Methods/Principal Findings:**

Using empirical data on the current patterns of freshwater fish biodiversity for rivers of peninsular India, we show here how the spatial changes alone under an archetypal IBWT project will (1) reduce freshwater fish biodiversity system-wide, (2) alter patterns of local species richness, (3) expand distributions of widespread species throughout peninsular rivers, and (4) decrease community richness by increasing inter-basin similarity (a mechanism for the observed decrease in biodiversity). Given the complexity of the IBWT, many paths to partial or full completion of the project are possible. We evaluate two strategies for step-wise implementation of the 11 canals, based on economic or ecological considerations. We find that for each step in the project, the impacts on freshwater fish communities are sensitive to which canal is added to the network.

**Conclusions/Significance:**

Importantly, ecological impacts can be reduced by associating the sequence in which canals are added to characteristics of the links, except for the case when all 11 canals are implemented simultaneously (at which point the sequence of canal addition is inconsequential). By identifying the fundamental relationship between the geometry of riverine networks and freshwater fish biodiversity, our results will aid in assessing impacts of IBWT projects and balancing ecosystem and societal demands for freshwater, even in cases where biodiversity data are limited.

## Introduction

Conflicts between the use of natural resources and conservation of biodiverse ecosystems are increasing worldwide as human population growth intensifies the demand for basic rights, most importantly the reliable access to fresh water [1], [2], [3], [4], [5]. Uncertainty in the timing, amount, and distribution of precipitation under a changing climate compound these conflicts [6]. Large-scale interbasin water transfer (IBWT) projects are commonly proposed as solutions to water distribution and supply problems [4], [7], [8]. However, changes to riverine network connectivity have been shown to affect population growth rates [9], population persistence [10], and patterns of biodiversity [11], [12].

**Figure 1:**
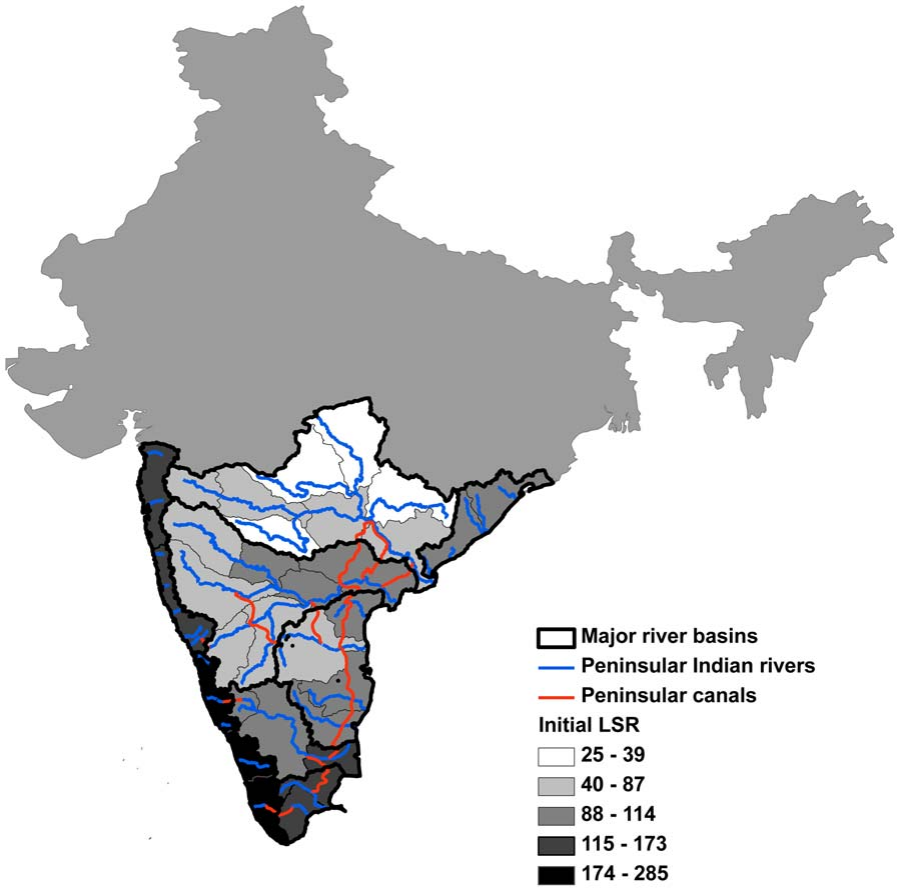
Map of peninsular India. Showing the 8 major river basins (thick black lines), 31 sub-basins (thin black or gray lines), major rivers (blue lines), 11 proposed canals under India's Interlinking of Rivers Programme interbasin water transfer plan (IBWT; red lines), and the initial local species richness (LSR; grey shading).

In IBWT projects, canals restructure river connectivity; such reconfigurations likely alter the stability and identity of riverine communities [10], [13], [14], [15], [16], [17]. One of the most ambitious IBWT plans is India's program to restructure the connectivity of the country's major rivers [8], [18]. Under the peninsular component of the project, 2245 km of canals are proposed to transfer water ‘surpluses’ from northern rivers to ‘deficit’ rivers in the south [18] (Fig. 1). Feasibility assessments for such large-scale projects are complex, involving elements of ecosystem, political, social, and economic sciences. Economic considerations often take precedence [19], while ecological consequences receive little attention [7], [19] partly because of mismatches between scales of biological data availability and the scale of the IBWT projects. In addition to the spatial complexity of India's IBWT project, rivers of peninsular India represent a global biodiversity hotspot for freshwater fish [20], emphasizing the tension between ecosystem consequences of an IBWT and a responsibility to provide reliable access to fresh water for a growing human population [19].

Spatial network structure is emphasized as a principal control on patterns of diversity [21], [22], with increasing connectivity among communities leading to higher local diversity for a given migration rate [21], [23]. Because connectivity is so critical to species diversity and biogeography, consideration of changes in the relative isolation of riverine networks (which may have been isolated over geological time) may provide a strategy for controlling impacts of IBWTs [11].

Here, we investigate how restructuring river connectivity via canals may influence patterns of species' distribution across India's peninsular rivers. We consider four aspects of freshwater fish biodiversity: system-wide changes in the total species richness (TSR, or γ diversity), changes in geographic patterns of local species richness (LSR, or α diversity), changes in the rank-occupancy distribution across species and changes in between-community richness (β diversity; calculated as 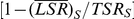 [24] where LSR is averaged across all sub-basins at each linking step *s*). To evaluate the potential effects of network restructuring on existing fish communities, we assess changes to biodiversity within the first 130 generations after sequential implementation of each canal under each strategy, where one generation represents the lifespan of an ‘average’ fish in the community.

## Methods

### River and watershed maps

We digitized maps from the Watershed Atlas of India (Central Groundwater Board, Ministry of Water Resources), dividing all peninsular rivers from the Godavari south into 31 major sub-basins mapped at the 1∶250000 scale (Fig. 1, Table 1). This region includes all but the northernmost canal link in the ‘Peninsular component’ of the IBWT [canals digitized from the National Water Development Agency maps (http://nwda.gov.in)].

**Table 1:** Number of sub-basins in each river basin.

River Basin	Number of sub-basins
Bhatsol	2
Cauvery	3
Godavari	8
Krishna	7
Pennar	4
Periyar	3
Vaippar	2
Vamsadhara	2
Total	31

From the Watershed Atlas of India, Central Groundwater Board, Ministry of Water Resources; cgwb.gov.in/watershed/basinsindia.html (accessed 22 October 2008); see also Fig. 1.

### Fish distribution data

We constructed a database of 4915 records of 457 freshwater fish species in river sub-basins across peninsular India. Locations of freshwater fish species were obtained from published and unpublished literature, species lists, online databases and reports (Table S1). We used multiple search engines (including Web of Science and Google), searching on combinations of river and tributary names, and additionally searched for cited records within each reference. In addition to distribution data contained in literature and reports, we queried museum and specimen databases for freshwater fish records in peninsular India: FishBase (www.fishbase.org; accessed 10 October 2008); Global Biodiversity Information Facility [www.gbif.org; accessed 03 October 2008 and included the following 3 collections accessed through GBIF data portal: IndOBIS, Indian Ocean Node of OBIS (http://data.gbif.org/datasets/resource/1471/03/10/2008); Biological Collection, National Institute of Oceanography, Goa, India (http://data.gbif.org/datasets/resource/1472/03/10/2008); and BoGART (http://data.gbif.org/datasets/resource/1087/03/10/2008)]. Nomenclature was checked against the California Academy of Sciences *Catalog of Fishes* [http://research.calacademy.org/ichthyology/catalog/fishcatsearch.html], ITIS [http://www.itis.gov/index.html], or FishBase [http://www.fishbase.org/search.php]. Where the nomenclature differed between sources, we followed the *Catalog of Fishes*. Fish were classified into habitat type (marine/brackish/secondary freshwater/freshwater) using the *Catalog of Fishes*, and we retained records of freshwater and secondary freshwater species only. Of 4915 database records, 949 were of ‘secondary freshwater’ - associated species (122 of 457 species). FishBase reports 801 species of freshwater fish in all of India, including the Ganges and Brahmaputra rivers (which are not considered here; www.fishbase.org; accessed 10 October 2008).

Fish were assigned to a river locality in one of two ways when latitude and longitude were not provided. If a map of the study area was given, this was matched within a GIS to determine the locality. When only descriptive information about sampling location was given in the text (e.g., names of nearby towns and cities, minor tributary names, names of nearby dams), we determined sampling locality within the Google Earth program, where rivers and their tributaries could be clearly seen, combined with place names and other geographic information. Locations that could not be matched with certainty were excluded (only 67 of 4915 total records).

### Species distribution modeling

We used the program MAXENT [25] to generate one potential distribution map for each species with >1 record in the database (including locations where they may occur but were not recorded), in relation to a set of 8 covariates that we hypothesized would be important for large-scale patterns of freshwater fish distribution [26]: annual average temperature [calculated from monthly average WorldClim data (30 arc-sec) [27]]; mean annual runoff [28], latitude and longitude at sub-basin center; sub-basin area [km2; Albers equal area projection]; flow direction [sub-basins were classified as draining to the east (Bay of Bengal/Indian Ocean) or the west (Arabian Sea)]; whether presence records were in coastal or inland sub-basins; sub-basin (Strahler) order [29], assigned order 1 (headwaters) to 4 (outlet); and identity of major river [specifying the major river associated with each sub-basin allows for endemism, a recognized feature of Indian rivers, especially those of the Western Ghats [20]]. Data were summarized for each of the 31 major sub-basins. By using this method to approximate biogeographic patterns of freshwater fish in under- and un-sampled sub-basins, we assume: (1) records are derived from a random sample of the entire landscape, (2) environmental variables at the presence locations represent favorable habitat for a given species, and (3) the distribution with maximum entropy describes the process by which presences are recorded in our database. We further assume (1) no temporal variation in species distribution, and (2) unbiased (or random) detection and inclusion of species in the database used for analysis. We used the default settings for convergence threshold, maximum iterations, and regularization, and chose a threshold value to assign presence to a river sub-basin which resulted in a closely matched distribution of species compared with published estimates of patterns of biodiversity in major rivers of peninsular India [30], [31], and of the observed LSR when the data were summarized for the 8 major peninsular river basins (i.e., the Bhatsol, Cauvery, Godavari, Krishna, Pennar, Periyar, Vaippar, Vamsadhara; R^2^ = 0.75).

### Application of a neutral model

Using this predicted distribution of fish biodiversity, we applied a neutral model [12], [22] to the sub-basins under different levels of implementation of the IBWT plan, including the case when no canals are implemented (see *Alternative strategies for canal implementation* section, below), to characterize impacts of the proposed IBWT canal links on freshwater fish biodiversity and biogeography. Neutral models are useful conservation tools [32] because of their ability to generate realistic patterns of biodiversity to evaluate biogeographic patterns of diversity and abundance, without requiring detailed data on species' interactions. This approach provides an efficient way to assess changes in biodiversity patterns driven by neutral processes (e.g., ecological drift [23]) operating within post-IBWT network geometries.

Habitat capacity was defined to be proportional to river length-weighted mean annual runoff. Nodes associated with outflow to the ocean were assigned zero habitat capacity. The four model parameters: the average number of new species introduced in each generation (θ), the mean habitat capacity of each river reach, and the movement parameters (*p* and *u*) which describe the shape of the ‘2Dt’ dispersal kernel [33], where dispersal distance (*x*) was distributed according to:
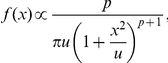
were estimated by fitting the model-estimated local species richness to the empirically-derived local species richness for the Cauvery basin (see also [12] for application and evaluation of the 2Dt dispersal model). The Cauvery basin was used to fit the model parameters because it had the highest occupancy data density, making it possible to resolve species richness at a finer scale than was possible over the entire study area. We find the best-fit parameter estimates by an iterative process of sequential one-dimensional optimizations of each parameter, where all remaining parameters are held at their current best-fit value, because significant stochasticity inherent to the modeling framework precluded use of downhill simplex or other, more sophisticated, multidimensional procedures.

**Figure 2:**
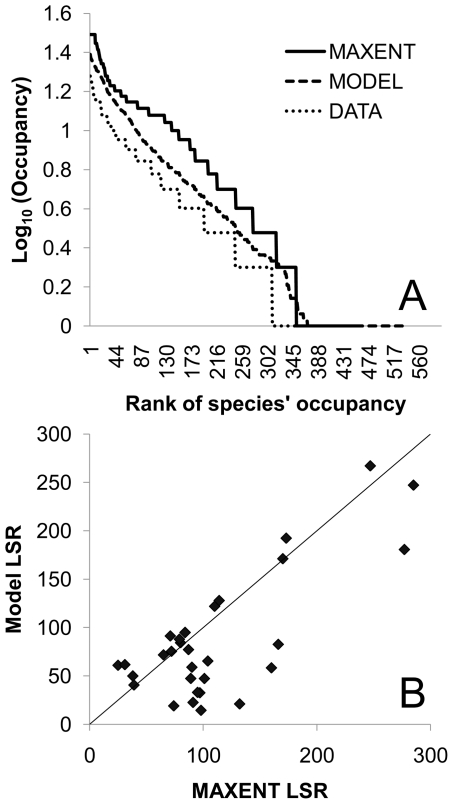
Plots of the model fits to the data used for the analysis. Panel A: rank-occupancy for the empirical freshwater fish data (dotted line), neutral model (dashed line; averaged over 120 simulation runs of the rank-occupancy after 130 generations), and the MAXENT-derived rank-occupancy distribution data (solid line). Panel B: relationship between the local species richness (LSR) from the neutral model (y-axis) and the MAXENT-derived distribution data (x-axes). The solid line is the 1∶1 line.

We present comparison of the MAXENT, and model-derived occupancy and LSR for the 31 sub-basin peninsular river networks (Fig. 2 A,B). While the model provided close correspondence with uncommon species (Fig. 2A, dashed line), the intermediate predicted values for occupancy across all ranks are reasonable, given our expectations of under-sampled sub-basins (in the database records; Fig. 2A, dotted line) and over-prediction of occupancy (resulting from the MAXENT model; Fig. 2A, solid line). The model performed well on predicting LSR for the MAXENT-derived data (R^2^ = 0.593; Fig. 2B).

### Alternative strategies for canal implementation

We consider two alternatives for sequential implementation of the 11 canals in the peninsular plan. First, we sum the expected economic benefit ([34]: Table 3.7) and divide by the cost (in 2003–04 dollars [34]: Table 3.5) for each canal separately, and order canals from lowest to highest cost-to-benefit ratio. To contrast this ‘economic’ strategy, we develop an ‘ecological’ linking strategy by ranking each canal according to factors which should minimize changes in biodiversity. Under this ecological linking strategy, we ranked canals in the order of least to most expected impact on biodiversity. The factors used to calculate this expected impact were based on simulation results [12]; we expect that the largest ecological impacts would come from: canals which connect more than 2 sub-basins (with the potential to link an increasing number of different communities), canals added between sub-basins at different positions in the river network (which harbor different communities), canals which connect rivers that differ greatly in total river basin areas (and proportionally larger differences in total community size), and shorter canals (which would facilitate exchange of species). We ran 120 replicate simulations for every canal linking step; we were computationally limited to storing only 13 ‘snapshots’ of data, taken every 10 generations during each simulation run, and thus use the first 130 generations after sequential implementation of each canal for our analyses.

### Quantifying changes to peninsular river connectivity

**Figure 3:**
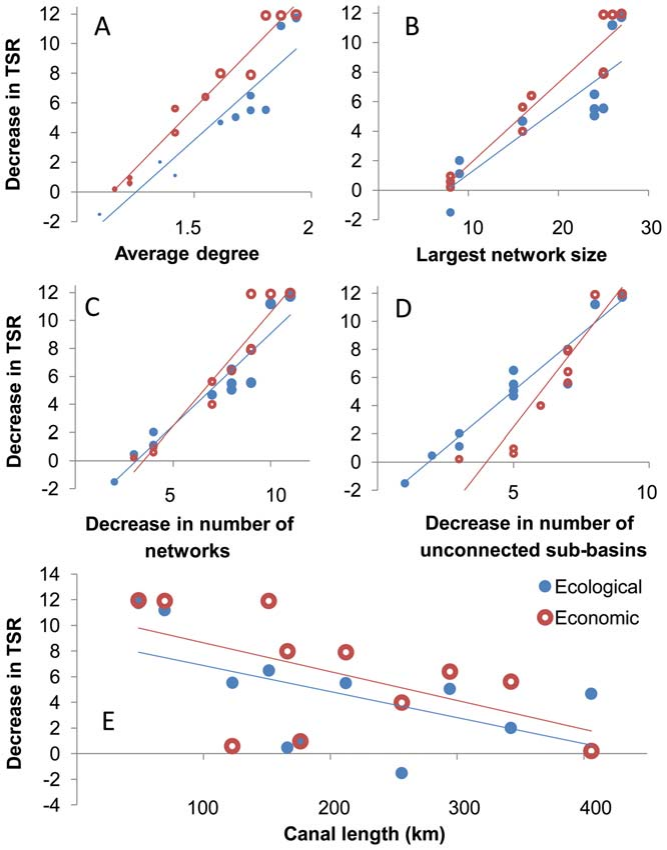
System connectivity underlies the loss of species (TSR, total species richness) for both the economic (open red circles) and the ecological (closed blue circles) linking strategies. This relationship holds for different measures of connectivity. Average degree (panel A; size of circles relative to the average number of connected sub-basins of the peninsular river networks, which increase as unconnected rivers are linked via canals), largest network size (panel B; size of circles relative to the average degree), decrease in the number of river networks (panel C; size of circles relative to the average degree), and number of networks consisting of a single sub-basin (panel D; size of circles relative to the average degree).

We characterize the change in system connectivity at each linking step using two metrics: (1) size and (2) degree. A river is comprised of ≥1 sub-basin, and each of the 31 sub-basins of the peninsular India river system may be hydrologically connected to other sub-basins via river segments or the addition of a canal; a *network* is this set of connected sub-basins. The *network size* is the number of connected sub-basins in a network; we report the average size across all networks at each linking step (Fig. 3B). A sub-basin's *degree* is comprised of the number of neighboring sub-basins that are connected to it via rivers or canals; we report the average degree, averaged across all 31 sub-basins of peninsular India for each linking step (Fig. 3A). We used analysis of covariance to investigate the relationship among the linking strategies (economic, ecological), connectivity metrics, and the change in total species richness (TSR).

## Results and Discussion

Increasing connectivity under the IBWT will have serious implications for the biodiversity of peninsular rivers, which have been isolated in geologic time and are recognized as a global ‘hotspot’ for fish biodiversity [20], [30]. Although any single IBWT link had insignificant consequences for TSR at the subcontinent scale (confirmed in theoretical work [12]), the sequential addition of canal links facilitated dispersal among previously isolated river networks, decreasing biodiversity across peninsular rivers. Characterizing the sequential modifications to the riverine network using connectivity metrics demonstrates that adding canal links changes the network geometry, which leads to biodiversity loss (Fig. 3A–D). The ecological and economic strategies determined the mean change in TSR, as a function of both the change in the average number of connected sub-basins (strategy: F(1,18) = 8.16, p = 0.010; average number of connected sub-basins: F(1,18) = 211.43, p<0.001; Fig. 3A), and the decrease in the number of hydrologically isolated sub-basins (strategy: F(1,18) = 6.97, p = 0.016; number of river networks: F(1,18) = 177.74, p<0.001; Fig. 3C) at each linking step; the interaction term was significant for only the decrease in the number of unconnected sub-basin river networks at each linking step (interaction model: strategy×number of unconnected river sub-basins: F(1,18) = 6.80; p = 0.018; Fig. 3D). Biodiversity losses were sensitive to small changes in connectivity, and to increases in the length of a canal linking sub-basins (Fig. 3E). Notably, the shortest canals will link the highly biodiverse rivers of the west-flowing rivers originating in the Western Ghats to east-flowing rivers, connecting rivers which have been separated in geologic time (120–130 m.y.a.) and are characterized by high endemism [20]. As a result, the loss in TSR is greatest for the canals linking western Ghats' rivers to the much larger Krishna and Cauvery rivers (Fig. 3E; canals <100 km in length). Although shorter canals facilitate the spread of species among sub-basins, the length of a canal alone does not predict the loss of species from the system (panel 2E; strategy: not significant).

**Figure 4:**
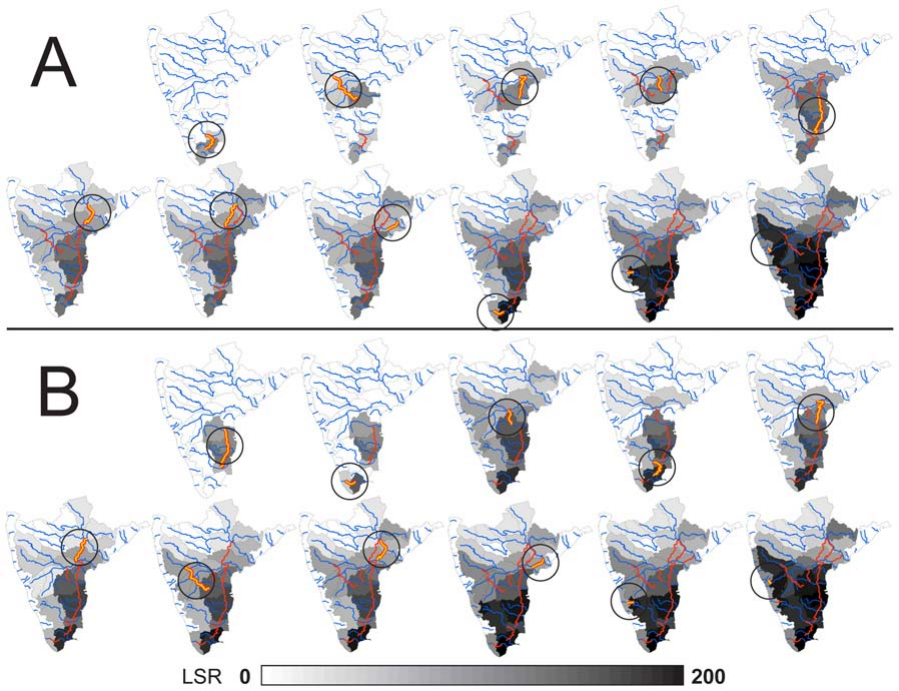
Change in local species richness (LSR) for each of the 31 river sub-basins as each canal is implemented. We show results from both the the economic (A) and ecological (B) linking strategies. Sub-basins are outlined in gray, major rivers in blue, and canals are indicated in red; the link added in each step is in yellow. Darker shades indicate greater increase in LSR (relative to the LSR after 130 generations with no canal links implemented). All other symbols follow Fig. 1.

**Figure 5:**
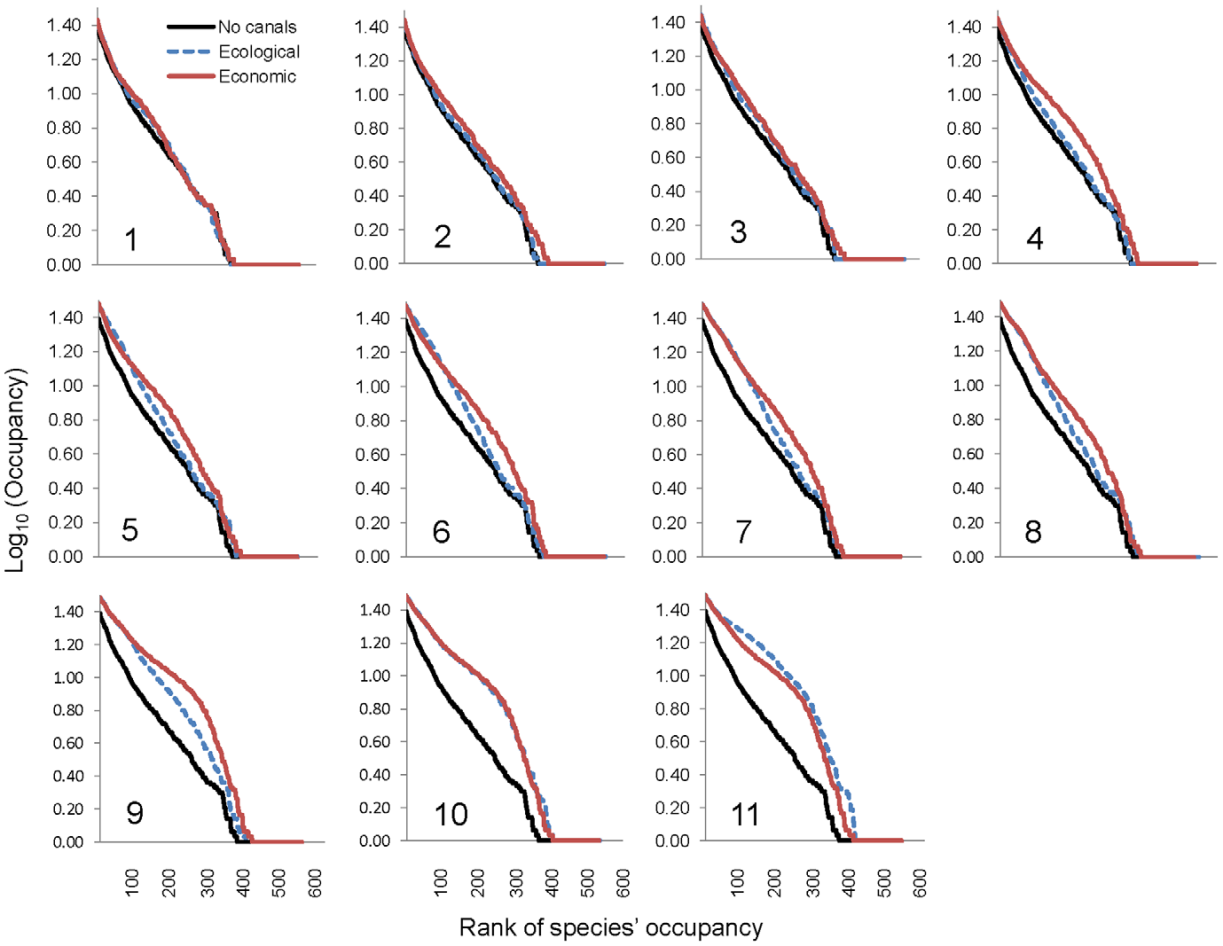
Average rank (of 130 generations) versus occupancy after each canal link is implemented. Note log scale of the y axis. Each of the 11 linking steps is represented by one plot. The lower, black line is the rank-occupancy under the no linking step; the solid red line is the resulting occupancy after each link is added under the ‘economic’ linking strategy, and the blue dotted line is the resulting occupancy after each link is added under the ‘ecological’ linking strategy. Each panel is an implementation step, where the canal added under the ecological strategy at a given step is not the same as the canal added in the economic strategy (compare canal implementation in Fig. 3).

**Figure 6:**
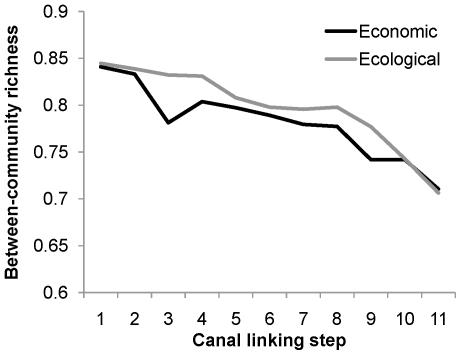
Between-community diversity for each linking strategy. The change in between-community diversity (i.e., β diversity) at each linking step is generally smaller for the ecological than the economic linking strategies. Note that the canal added at steps 1–9 (but not 10 or 11) differ between strategies (compare canal implementation in Fig. 3).

As canals are added, LSR increases in each connected sub-basin, with the largest increases occurring in those directly linked (Fig. 4). As the first canals are implemented, widespread species are particularly advantaged by the increased connectivity, but as the network becomes increasingly connected, intermediate species also increase in frequency and spatial extent within the restructured river network (Fig. 5). Widespread species have a numerical advantage over rare species for colonization with increasing connectivity because they occupy a greater proportion of the system prior to canal linking. For each canal addition, local communities become generally more diverse (Fig. 4), but also become increasingly similar to one another in their species compositions (Fig. 6). That is, the implementation of these canals may bring about increases in local diversity at the expense of diversity at the peninsular scale.

### Can biodiversity be balanced with water supply?

Satisfying an increasingly thirsty world while protecting biodiversity is a daunting task [1], [2], [3], [4], [5]; our results demonstrate that the IBWT plan will decrease biodiversity and change the biogeography of peninsular rivers by altering patterns of riverine network connectivity. Not only does spatial connectivity matter, but the actual sequence in which rivers are linked up affects biodiversity patterns at continental scales (unless all 11 canals are implemented simultaneously). Compared to the ‘economic’ linking strategy, the ‘ecological’ strategy affords (1) a reduced rate of biodiversity losses as network connectivity increases (Fig. 3), (2) smaller spatial changes to LSR as each canal is implemented (Fig. 4), (3) slower increases in the occupancy of widespread and mid-rank species (Fig. 5), and (4) slower decline in between-community diversity (Fig. 6). Because the effect on LSR of each canal is primarily local (though changes resonate across connected sub-basins; Fig. 4), decision-makers can evaluate whether the impacts on biological communities are outweighed by the importance of each canal. For example, there are large changes in occupancy and spatial patterns of LSR when sub-basins in the western Ghats (with higher endemicity [20]) are connected to sub-basins of the larger, east-draining rivers (with more widespread species; Fig. 4). The actual impacts of the IBWT plan will also depend on the amounts of transferred water, asymmetric competition, larvivory and other trophic interactions, and differential abilities of species to benefit from habitats created by and accessible via the canals [11]; nonetheless, our estimates of species loss and changes in fish biodiversity patterns describe the minimum impacts of an IBWT project on biodiversity and biogeography, fundamentally due to the altered connectivity, on top of which other biological realism can be added and explored.

## Supporting Information

Table S1 **References used to develop our database of freshwater fish species locations.**
(DOCX)
