# Supplementary material for: Interbasin Water Transfer, Riverine Connectivity, and Spatial Controls on Fish Biodiversity
Source: PLoS One. 2012 Mar 28;7(3):e34170. doi: 10.1371/journal.pone.0034170 (PMC3314595; doi:10.1371/journal.pone.0034170)
Supplement: Table S1 — References used to develop our database of freshwater fish species locations. (DOCX) [file pone.0034170.s001.docx]

1. Adhikari, S., Lopa Ghosh, B.S. Giri, and S. Ayyappan. 2009. Distributions of metals in the food web of fishponds of Kolleru Lake, India. Ecotoxicology and Environmental Safety 72: 1242-1248.
2. Agarwal, R., R. Kumar, and J.R. Behari. 2007. Mercury and lead content in fish species from the River Gomti, Lucknow, India, as biomarkers of contamination. Bulletin of Environmental Contamination and Toxicology 78: 118-122.
3. Ajithkumar, C.R., C.R. Biju, K. Raju Thomas, and P.A. Azeez. 2001. On the fishes of Puyankutty river, Kerala, India. Zoos’ Print Journal 16: 467-469
4. Ajithkumar, C.R., K. Rema Devi, K. Raju Thomas, and C.R. Biju. 1999. Fish fauna, abundance and distribution in Chalakudy river system, Kerala. Journal of the Bombay Natural History Society 96: 244-254.
5. Ajmal, M., R. Kahn, and A.U. Khan. 1987. Heavy metals in water, sediments, fish and plants of river Hindon, U.P., India. Hydrobiologia 148: 151-157.
6. Ajmal, M., M. A. Khan, and A.A. Nomani. 1985. Distribution of heavy metals in plants and fish of the Yamuna River (India). Environmental Monitoring and Assessment 5: 361-367.
7. Ali, S., K.V. Gururaja, and T.V. Ramachandra. 2005. *Schisture niligriensis* (Menon) in Sharavathi River Basin, Western Ghats, Karnataka. Zoos’ Print Journal 20: 1784-1785.
8. Ali, A.P. H., and G. Prasad. 2007. Bionomics of a critically endangered and endemic catfish, *Horabagrus nigricollaris* from its type locality in Kerala. Journal of the Bombay Natural History Society 104: 167-171.
9. Anoop, K.R., and S.A. Hussain. 2005. Food and feeding habits of smooth-coated otters (*Lutra perspicillata*) and their significance to the fish population of Kerala, India. Journal of Zoology 266: 15-23
10. Arun, L.K., C.P. Shaji, and P.S. Easa. 1995. Record of new fishes from Periyar Tiger Reserve. Journal of the Bombay Natural History Society 93: 103-104.
11. Arunachalam, M. 2000. Assemblage structure of stream fishes in the Western Ghats (India). Hydrobiologia 430: 1-31.
12. Arunachalam, M., and J.A. Johnson. 2002. A new species of Puntius (Hamilton) (Pisces: Cyprinidae) from Kalakad-Mundanthurai Tiger Reserve, Tamil Nadu, India. Journal of the Bombay Natural History Society 99: 474-480.
13. Arunachalam, M., J.A. Johnson, A. Manimekalan, and S. Sridhar. 1999. New record of *Heteropneustes microps* (Gunther) (Siluridae: Heteropneustidae) from the Western Ghats rivers, India. Journal of the Bombay Natural History Society 96: 330-332.
14. Arunachalam, M., J.A. Johnson, A. Manimekalan, A. Sankaranarayanan, R. Soranam, P. Sivakumar, and M. Muralidharan. 2005. Extension of range of *Puntius arulius arulius* (Jerdon) in various streams in Thungabadra river basin. Journal of the Bombay Natural History Society 102: 343-344.
15. Arunuachalam, M., J.A. Johnson, and P.N. Shanthi. 1999. A new record of the marine puffer fish genus, *Chelonodon* (Tetraodontiformes, Tetraodontidae) from freshwater habitat of Western Ghats, India. Acta Zoologica Taiwanica 10: 11-14.
16. Arunachalam, M., J.A. Johnson, and R. Soranam. 1998. New record of *Puntius melanampyx* (Cypriniformes: Cyprinidae) and *Microphis cuncalus* (Syngnathoformes: Syngnathidae) from Karnataka, India. Journal of the Bombay Natural History Society. 95: 128-129.
17. Arunachalam, M., and M. Muralidharan. 2008. Description of a new species of the genus *Psilorhynchus* (Teleostei: Psilorhynchidae) from a Western Ghat stream in south India. Raffles Bulletin of Zoology 56: 405-414.
18. Arunachalam, M., and M. Muralidharan. 2009. *Nemacheilus stigmofasciatus*, a new species of nemacheiline loach (Cypriniformes: Balitoridae) from the Western Ghats, India. Journal of Threatened Taxa 1: 147-150.
19. Arunachalam, M., and A. Sankaranarayanan. 1999. New records of fishes from Gadana River, Kalakad Mundanthurai Tiger Reserve, Tamil Nadu. Journal of the Bombay Natural History Society 96: 336-337.
20. Arunachalam, M., and A. Sankaranarayanan. 1999. Fishes of Gadana River in Kalakkad Mundanthurai Tiger Reserve. Journal of the Bombay Natural History Society 96: 232-238.
21. Arunachalam, M., A. Sankaranarayanan, J.A. Johnson, A. Manimekalan, and R. Soranam. 2000. New records of fishes from the Western Ghats of Maharashtra. Journal of the Bombay Natural History Society 97: 292-294.
22. Arunachalam, M., A. Sankaranarayanan, J.A. Johnson, A. Manimekalan, R. Soranam, P.N. Shanthi, and C. Vijaykumar. 2000. Fishes of Nambiyar River, Kalakad-Mundanthurai Tiger Reserve, Tamil Nadu. Journal of the Bombay Natural History Society 97: 153-154.
23. Arunachalam, M., A. Sankaranarayanan, A. Manimekalan, R. Soranam, and J.A. Johnson. 1999. New record of *Salmostoma sardinella* (Pisces: Cyprinidae) from Mondai stream, Maharashtra. Journal of the Bombay Natural History Society 96: 162-163.
24. Arunachalam, M., A. Sankaranarayanan, A. Manimekalan, R. Soranam, and J.A. Johnson. 2002. Fish fauna of some streams and rivers in the Western Ghats of Maharashtra. Journal of the Bombay Natural History Society 99: 337-340.
25. Arunachalam, M., A. Sankaranarayanan, R. Soranam, J.A. Johnson, and A. Manimekalan. 1999. New record of *Stigmatogobius oligactis* (Bleeker) from India. Journal of the Bombay Natural History Society 96: 167-168.
26. Arunachalam, A., R. Sarmah, D. Adhikari, M. Majumder, and M.L. Khan. 2004. Anthropogenic threats and biodiversity conservation in Namdapha nature reserve in the Indian Eastern Himalayas. Current Science 87: 447-454.
27. Asokan, R., and P. Shahul Hameed. 1992. Distribution of natural radionuclide ^40^K in biotic and abiotic components of the Cauvery river system, Tiruchirapalli, India. Journal of Biosciences 17: 491-427.
28. Babu, K.K.S., and Nayar, C.K.G. 2004. A new species of the blind fish *Horaglanis* (Menon) (Siluroifea: Claridae) from Parappukara (Trichur District) and a new report of *Horaglanis krishnai* Menon from Ettumanur (Kottayam District), Kerala. Journal of the Bombay Natural History Society 101: 296-299.
29. Babu Rao, M. 1977. Extension of distribution of *Thrissina boelama* (Forksal) (Pisces: Clupeiformes: Engraulidae) with remarks on its taxonomic characters. Journal of the Bombay Natural History Society 74: 365-366.
30. Babu Rao, M., and G.M. Yazdani. 1977. Occurrence of cobitid genus *Botia* (Gray) in the Western Ghats of India. Journal of the Bombay Natural History Society 74: 367-368.
31. Balasanundaram, C., R. Arumugam, and P. B. Murugan. 2001. Fish diversity of Kolli Hills, Western Ghats, Salem District, Tamil Nadu. Zoos' Print Journal 16: 403-406.
32. Banarescu, P.M., and B. Herzig-Straschil. 1995. A revision of the species of the *Cyprinion macrostomum*-group (Pisces: Cyprinidae). Annalen Naturhistorischen Museums in Wien 97: 411-420.
33. Banerji, S.R., M.L. Singh, S.K. Thakur, and N.K. Thakur. 1980. Khulnawa - a special fishing device for minnows in the river Ganga at Patna (Bihar). Journal of the Bombay Natural History Society 77: 351-353.
34. Barman, R.P. 1985. On a new cyprinid fish of the genus *Barilius* (Hamilton) (Pisces: Cyprinidae) from Arunachal Pradesh, India. Journal of the Bombay Natural History Society 82: 170-174.
35. Barman, R.P. 1986. A new freshwater fish of the genus *Barilius* (Hamilton) (Pisces: Cyprinidae) from West Bengal, India. Journal of the Bombay Natural History Society. 83: 171-172.
36. Barman, R.P, S.S. Mishra, and A. Das. 2006. On the occurrence of *Cypselurus poecilopterus* (Valenciennes) (Pisces: Exocoetidae) in Indian Waters. Journal of the Bombay Natural History Society 103: 109-110.
37. Batvaru, B., S. Kamala-Kannan, K. Shanthi, R. Krishnamoorthy, K.J. Lee, and M. Jayaprakash. 2008. Heavy metals in two fish species (*Carangoidel malabaricus* and *Belone stronglurus*) from Pulicat Lake, North of Chennai, Southeast Coast of India. Environmental Monitoring and Assessment 145: 167-175.
38. Bhagowati, A.K., and B.K. Biswas. 1991. Record of *Colisa labiosa* (Day) (Pisces: Belontidae) from India. Journal of the Bombay Natural History Society 88: 293.
39. Bhargava, R.N. 1974. On a new record of Murrel, *Channa leucopunctatus* (Sykes) from Rajasthan. Journal of the Bombay Natural History Society 72: 152-153.
40. Bhat, A. 2003. Diversity and composition of freshwater fishes in river systems of Central Western Ghats, India. Environmental Biology of Fishes 68: 25-38.
41. Bhat, A. 2004. Patterns in the distribution of freshwater fishes in rivers of Central Western Ghats, India and their associations with environmental gradients. Hydrobiologia 529: 83-97.
42. Bhusari, B.V. 1975. Biology and fishery of *Pseudosciaena sina* (Cuvier) at Ratnagiri, South Maharashtra. Journal of the Bombay Natural History Society 72: 357-367.
43. Biju, C.R., K. Raju Thomas, and C.R. Ajithkumar. 1998. *Sicyopterus griseus* (Day) from Periyar river, Kerala. Journal of the Bombay Natural History Society 95: 351-352.
44. Biju, C.R., K. Raju Thomas, and C.R. Ajithkumar. 1998. *Glyptothorax lonah* (Sykes) - an addition to the ichthyofauna of Kerala. Journal of the Bombay Natural History Society 95: 519-520.
45. Biju, C.R., K. Raju Thomas, and C.R. Ajithkumar. 1998. Distribution of fish in the Manjeswaram river, Kasaragod (Kerala). Journal of the Bombay Natural History Society 96: 159-161.
46. Biju, C.R., K. Raju Thomas, and C.R. Ajithkumar. 1998. *Macrospinosa cuja* (Hamilton-Buchanan): a new record from Kerala. Journal of the Bombay Natural History Society 96: 166-167.
47. Biju, C.R., K. Raju Thomas, and C.R. Ajithkumar. 1999. Fishes of Parambikulam Wildlife Sanctuary, Palakkad District, Kerala. Journal of the Bombay Natural History Society 96: 82-87.
48. Biju, C.R., K. Raju Thomas, and C.R. Ajithkumar. 1999. Occurrence of *Tetraodon travancoricus* in the Chalakudy, Periyar and Kechery rivers, Kerala. Journal of the Bombay Natural History Society 96: 161.
49. Biju, C.R., K. Raju Thomas, and C.R. Ajithkumar. 1999. Distribution of freshwater fishes in the Uppala river, Kasargod District, Kerala. Journal of the Bombay Natural History Society 96: 334-335.
50. Biju, C.R., K. Raju Thomas, and C.R. Ajithkumar. 1999. Range extension of *Osteobrama cotio peninsularis* to Kerala. Journal of the Bombay Natural History Society 96: 481-482.
51. Brahmane, M.P., M.K. Das, M.R. Sinha, V.V. Sugunan, A. Mukherjee, S.N. Singh, S. Prakash, P. Maurye, and A. Hajra. 2006. Use of RAPD fingerprinting for delineating populations of Hilsa shad *Tenualosa ilisha* (Hamilton, 1822). Genetics and Molecular Research 5: 643-652.
52. Chandanshive, N.E., S.M. Kamble, and B.E. Yadav. 2007. Fish fauna of Pavana River of Pune, Maharastra. Zoos' Print Journal 22: 2693-2694.
53. Chatterji, A. 1980. The relative condition factor and length-weight relationship of a freshwater Carp, *Labeo gonius* (Hamilton) (Cyprinidae: Teleostei). Journal of the Bombay Natural History Society 77: 435-443.
54. Chatterji, A., A.Q. Siddiqui, and A.A. Khan. 1978. Food and feeding habits of *Labeo gonius* (Hamilton) from the river Kali. Journal of the Bombay Natural History Society 75: 104-109.
55. Chaudhuri, B.L. 1912. Description of some new species of freshwater fishes from north India. Records of the Indian Museum 7:437-444.
56. Chauhan, T., K.K. Lal, V. Mohindra, R.K. Singh, P. Punia, A. Gopalakrishnan, P.C. Sharma, and W.S. Lakra. 2007. Evaluating genetic differentiation in wild populations of the Indian major carp, *Cirrhinus mrigala* (Hamilton–Buchanan, 1882): Evidence from allozyme and microsatellite markers. Aquaculture 269: 135-149.
57. Dahanukar, N., R. Raut, and A. Bhat. 2004. Distribution, endemism and threat status of freshwater fish in the Western Ghats of India. Journal of Biogeography 31: 123-136.
58. Datta, A.K., R.P. Barman, and K.C. Jayaram. 1987. On a new species of Kryptopterus (Pisces: Siluroidea, family: Siluridae) from Namdapha Wildlife Sanctuary, Arunachal Pradesh, India. Bulletin of the Zoological Survey of India 8: 29-31.
59. David, A. 1963. Studies on fish and fisheries of the Godavari and Krishna river systems. Part I & 2. Proceedings of the Indian National Academy of Science 33: 263-286.
60. Desai, V.R. 1973. Studies on fishery and biology of *Tor tor* (Hamilton) from river Narmada. Proceedings of the Indian National Academy of Science 39: 228-248.
61. Easa, P.S., and C.P. Shaji. 1996. Freshwater fishes of Pambar river, Chinnar Wildlife Sanctuary, Kerala. Journal of the Bombay Natural History Society 93: 304-305.
62. Easa, P.S., and C.P. Shaji.1997. Freshwater fish diversity in Kerala part of the Nilgiri Biosphere Reserve. Current Science 73: 180-182.
63. Ganasan, V., and R.M. Hughes. 1998. Application of an index of biological integrity (IBI) to fish assemblages of the rivers Khan and Kshipra (Madhya Pradesh), India. Freshwater Biology 40: 367-383.
64. Garg, R.K., R.J. Rao, and D.N. Saksena. 2007. Checklist of fishes of Ramsagar Reservoir, Datia District, Madhya Pradesh, India. Zoos' Print Journal 22: 2801-2803.
65. Ghate, H.V., V.M. Pawar., and B.E. Yadav. 2002. Note on a horned Cyprinoid fish *Schismatorhynchos* (*nukta*) *nukta* (Sykes) from the Krishna drainage, Western Ghats. Zoos' Print Journal 17: 830-831.
66. Ghate, H.V., and G.K. Wagh. 1991. First record of a belontid fish *Macropodus cupanus* (Valenciennes) from Pune, Maharashtra. Journal of the Bombay Natural History Society 88: 124-125.
67. Ghosh, S.K., and A.G. Ponniah. 2008. Freshwater fish habitat science and management in India. Aquatic Ecosystem Health and Management 11: 272-288.
68. Gopalakrishnan, A., P.M. Abdul Muneer, K.K. Musammilu, K.K. Lal, D. Kapoor, and V. Mohindra. 2006. Primers from the orders Osteoglossiform and Siluriform detect polymorphic microsatellite loci in sun-catfish, *Horabagrus brachysoma* (Teleostei: Bagridae). Journal of Applied Ichthyology. 22: 456–458.
69. Haniffa, M.A., M. Nagarajan, A. Gopalakrishnan, and K.K. Musammilu. 2007. Allozyme variation in a threatened freshwater fish, spotted murrel (*Channa punctatus*) in a South Indian River System. Biochemical Genetics 45: 363-374.
70. Harikumar, S., K.G. Padmanabhan, P.A. John, and K. Kortmulder. 1994. Dry-season spawning in a cyprinid fish of southern India. Environmental Biology of Fishes 39: 129-136.
71. Hegde, V.N. 2004. Occurrence of the bow-mouth guitar-fish *Rhina ancylostoma* (Bloch and Schneider 1801) off Versova, Mumbai, India. Journal of the Bombay Natural History Society 103: 111.
72. Hiware, C.J., and R.T. Pawar. 2004. Ichthyofauna of Paithan Reservoir (Nath Sagar Dam) in Aurangabad District of Marathwada Region, Maharashtra State, p. 209-214 *In* Ecology and Environment (B.N. Pandey and M.K. Jyoti, eds.). A.P.H. Publishing Corporation, New Delhi, India.
73. Hora, S. L. 1944. On the Malayan affinities of the freshwater fish fauna of peninsular India, and its bearing on the probable age of the Garo-Rajmahal gap. Proceedings of the National Institute of Sciences of India 10: 423-439.
74. Inasu, N.D. 1993. Sexual dimorphism of a fresh water puffer fish, *Tetraodon travancoricus* (Hora and Nair), collected from Trichur district, Central Kerala. Journal of the Bombay Natural History Society 90: 523-524.
75. Institute of Marine and Coastal Sciences, Rutgers University, South African Institute for Aquatic Biodiversity - Fish Collection (AfrOBIS).
76. Jameela Beevi, K.S., and A. Ramachandran. 2005. A new species of *Puntius* (Cyprinidae, Cyprininae) from Kerala, India. Journal of the Bombay Natural History Society 102: 83-85.
77. Jayaraj, E.G., D.S. Krishna Rao, S. Ravichandra Reddy, K. Shakuntala, and K.V. Devaraj. 1999. A new cyprinid fish of the genus *Salmostoma* (Swainson) from a tropical reservoir of south India. Journal of the Bombay Natural History Society 96: 113-115.
78. Jayaram, K.C. 1997. Massacre of mahsheer fish. Current Science 72: 901.
79. Jayaram, K.C. 1990. On two new species of the genus *Puntius* (Hamilton) from India (Pisces: Cyprinidae). Journal of the Bombay Natural History Society 87: 106-109.
80. Johsi, K.D. 1998. *Chagunius chagunio* (Hamilton-Buchanan) (Pisces: Cyprinidae): a new record from Kumaon Hills, Uttar Pradesh. Journal of the Bombay Natural History Society 95: 127-128.
81. Johsi, K.D., and P.C. Josi. 1996. *Puntius dukai* (Day) (Pisces: Cyprinidae) - a new record from Uttar Pradesh hills. Journal of the Bombay Natural History Society 93: 102.
82. Kamata, N.D. 1971. Algal food of *Aplocheilus blochii* (Arnold). Journal of the Bombay Natural History Society 71: 151-152.
83. Kar, D., A.V. Nagarathna, T.V. Ramachandra, and S.C. Dey. 2006. Fish diversity and conservation aspects in an aquatic ecosystem in northeastern India. Zoos’ Print Journal 21: 2308-2315.
84. Karamchandani, S.J., and P.K. Pandit. 1975. On the occurrence and breeding of *Labeo rohita* (Hamilton) in a section of Narbada river in Gujarat State. Journal of the Bombay Natural History Society 72: 215-217
85. Khan, H.A., and M.Y. Kamal. 1979. On a collection of fish from river Kosi (Bihar). Journal of the Bombay Natural History Society 76: 530-534.
86. Kumar, K.S., K. Kannan., O.N. Paramasivan, V.P. Shanmuga Sundaram, J. Nakanishi, and S. Masunaga. 2001. Polychlorinated dibenzo-*p*-dioxins, dibenzofurans, and polychlorinated biphenyls in human tissues, meat, fish, and wildlife samples from India. Environmental Science and Technology 35: 3448-3455.
87. Kurup, B.M., T.G. Manojkumar, and K.V. Radhakrishnan. 2005. *Salarias reticulatus* (Pisces: Blennidae), a new freshwater blenny from Chalakudy River, Kerala. Journal of the Bombay Natural History Society 102: 195-197.
88. Kurup, B.M., K.V. Radhakrishnan, and T.G. Manojkumar. 2004. Biodiversity status of fishes inhabiting rivers of Kerala (S. India) with special reference to endemism, threats and conservation measures. Proceedings of the Second International Symposium on the Management of Large Rivers for Fisheries 2: 163-182.
89. Malik, A., P. Ojha, and K.P. Singh. 2008. Distribution of polycyclic aromatic hydrocarbons in edible fish from Gomti River, India. Bulletin of Environmental Contamination and Toxicology 80: 134-138.
90. Manakadan, R. 1993. Occurrence of the freshwater grey mullet *Rhinomugil corsula* (Hamilton) at the Tungabhadra-Krishna confluence near Nandikotkur, Andhra Pradesh. Journal of the Bombay Natural History Society 90: 522-523.
91. Manakadan, R. 2003. Occurrence of the two-spot gourami *Trichogaster trichopterus* (Pallas) in Porur, Chennai, Tamil Nadu, India. Journal of the Bombay Natural History Society 102: 236.
92. Mandal, A., K.K. Lal, V. Mohindra, R.K. Singh, P. Punia, U.K. Chauhan, and W.S. Lakra. 2009. Evaluation of genetic variation in the clown knifefish, *Chitala chitala*, using allozymes, RAPD, and microsatellites. Biochemical Genetics 47: 216–234.
93. Mandy, T.J., and N.D. Inasu. 1998. Sexual dimorphism of a perch *Priacanthus hamrur* (Cuvier and Valenciennes). Journal of the Bombay Natural History Society 95: 132-133.
94. Manimekalan, A., and M. Arunachalam. 2002. Rediscovery of critically endangered air breathing catfish *Clarias dayi* (Hora) (Pisces: Claridae) at Mudumalai Wildlife Sanctuary, Tamil Nadu. Journal of the Bombay Natural History Society 99: 129-130.
95. Manimekalan, A., M. Arunachalam, and K. Rema Devi. 2005. New records and range extension of freshwater fishes to the Nilgiri Biosphere Reserve, South India. Journal of the Bombay Natural History Society 102: 344-345.
96. Manimekalan, A., and H.S. Das. 1998. *Glyptothorax davissinghi* (Pisces: Sisoridae): a new cat fish from Nilambur in the Nilgiri Reserve, South India. Journal of the Bombay Natural History Society 95: 87-91.
97. Manimekalan, A., and D.F. Singh. 1997. New record of *Schismatorhynchus* (*nukta*) *nukta* (Pisces: Cyprinidae) from Moyar river, Tamil Nadu. Journal of the Bombay Natural History Society 94: 170-171.
98. Manojkumar, T.G., and B. Madhusoodana Kurup. 2002. *Tor putitora* as an addition to the fish fauna of Peninsular India. Journal of the Bombay Natural History Society 101: 465.
99. Martin, P., M.A. Haniffa, and M. Arunachalam. 2000. Abundance and diversity of macroinvertebrates and fish in the Tamiraparani river, South India. Hydrobiologia 430: 59-75.
100. Menon, A.G.K. 1954. Further observations on the fish fauna of Manipur state. Records of the Indian Museum 52: 21-26.
101. Menon, A.G.K. 2004. Threatened fishes of India and their conservation. Zoological Survey of India, Kolkata, India. 170 pp.
102. Menon, A.G.K., and P.C. Jacob. 1996. *Crossocheilus periyarensis*, a new Cyprinid fish from Thanikkudy, Kerala, India. Journal of the Bombay Natural History Society 93: 62-63.
103. Menon, M.A.S. 1952. On a small collection of fish from Manipur. Records of the Indian Museum 52: 265-270.
104. Mercy, T.V.A., and E. Jacob. 2006. A new species of Teleostei: *Puntius pookodensis* (Cyprinidae) from Wayanad, Kerala, India. Journal of the Bombay Natural History Society 104: 76-78.
105. Mohanty, S.K. 1973. Further additions to the fish fauna of the Chilka Lake. Journal of the Bombay Natural History Society 72: 863-868.
106. Motwani, M.P., and A. David. 1957. Fishes of the River Sone with observations on the zoogeographical significance. Journal of the Zoological Society of India 9: 9-14.
107. Muneer, P.M.A., A. Gopalakrishnan, K.K. Lal, V. Mohindra. 2007. Population genetic structure of endemic and endangered yellow catfish, *Horabagrus brachysoma*, using allozyme markers. Biochemical Genetics 45: 637-645.
108. NG, H.H., and Z. Jaafar. 2008. A new species of leaf fish, *Nandus andrewi* (Teleostei: Perciformes: Nandidae) from northeastern India. Zootaxa 1731: 24-32.
109. Pandey, A.C. 1982. Effect of corticoid administration and osmotic disturbances on the adrenocortical homologue (AH) of a freshwater catfish, *Ompok bimaculatus* (Bloch). Mikroskopie 39:81-84.
110. Pandey, A.C. 1994. Evidence for general hypocalcemic hormone from the stannius corpuscles of the freshwater catfish, *Ompok bimaculatus* (Bloch). General and Comparative Endocrinology 94: 182-185.
111. Paralkar, V.K. 2006. Comments on the distribution of the flying barb *Esomus danricus*. Journal of the Bombay Natural History Society 103: 114.
112. Pardhasaradhi, A., and J.R.B. Alfred. 1981. A note on the biogeographical relictness of *Pillaia indica* Yazdani (Pillaidae: Mastacembeloidei). Journal of the Bombay Natural History Society 78: 175-177.
113. Pathani, S.S. 1980. The giant mahseers of Kumaun Himalayas with a recent rare record. Journal of the Bombay Natural History Society 77: 154-155.
114. Pethiyagoda, R, and M. Kottelat. 1994. Three new species of fishes of genera *Osteochilichthys* (Cyprinidae), *Travancoria* (Balitoridae) and *Horabargus* (Bagridae) from the Chalakudy River, Kerala, India. Journal of South Asian Natural History 1: 97-116.
115. Pethiyagoda, R. & Kottelat, M. 2005. A review of the barb of the *Punitius filamentosus* group (Teleostei: Cyprinidae) of southern India and Sri Lanka. Raffles Bulletin of Zoology Supplement 12:127-144.
116. Pillay, S.R. 1964. Maturation and spawning of the hilsa, *Hilsa ilisha* (Hamilton), of the Saurashtra Coast. Proceedings of the National Institute of Sciences of India 30: 8-14.
117. Pradhan, M.S., and D.F. Singh. 1984. First record of the freshwater grey mullet, *Rhinomugil corsula* (Hamilton) from Maharashtra. Journal of the Bombay Natural History Society 81: 202-204.
118. Prasad, G., and P.H. Anvar Ali. 2008. Morphology of the diet in the gut of threatened yellow catfish *Horabagrus brachysoma* (Gunther 1864) at two life stages. Fish Physiology and Biochemistry: 34: 385-389.
119. Radhakrishnan, K.V., and B.M. Kurup. 2005. Extension of range of *Nemacheilus keralensis* (Rita and Nalbant) and *Puntius ophicephalus* (Raj) to River Meenachil, Kerala (India). Journal of the Bombay Natural History Society 102: 236-237.
120. Radhakrishnan, K.V., and B. M. Kurup. 2006. Range extension of *Travancoria elongata* (Pethiyagoda and Kottelat) to River Periyar, Kerala, South India. Journal of the Bombay Natural History Society. 103: 113-114.
121. Raghavan, R., G. Prasad, P.H. Anvar Ali, and B. Pereira. 2008. Exotic fish species in a global diversity hotspot: observation from River Chalakudy, part of Western Ghats, Kerala, India. Biological Invasions 10: 37-40.
122. Raghaven, R., G. Prasad, P.H. Anvar Ali, and B. Pereira. 2008. Fish fauna of Chalakudy River, part of Western Ghats biodiversity hotspot, Kerala, India: patterns of distribution, threats and conservation needs. Biodiversity Conservation 17: 3119-3131.
123. Raghaven, R., G. Prasad, P.H. Anvar Ali, and L. Sujarittanonta. 2007. ‘Boom and bust fishery’ in a biodiversity hotspot – Is the Western Ghats losing its most celebrated native ornamental fish, *Puntius denisonii* (Day)? Current Science 92: 1671-1672.
124. Raizada, S.B. 1983. Occurrence of common carp (*Cyprinus carpio*) in trout waters. Journal of the Bombay Natural History Society 80: 430.
125. Raj, B.S. 1941. A new genus of schizothoracine fishes from Travancore, South India. Records of the Indian Museum 43:209-214.
126. Rajagopal, K.V., and V. Muddanna. 1972. Two unique methods of fishing for Cobitids in Tungabhadra River. Journal of the Bombay Natural History Society 69: 429-432.
127. Raju Thomas, K., C.R. Biju, and C.R. Ajithkumar. 1998. Rainbow trout (*Salmo gairdnerii*) in Anamalai Hills, Western Ghats. Journal of the Bombay Natural History Society 95: 350-351.
128. Raju Thomas, K., C.R. Biju and C.R. Ajithkumar. 1998. First report of *Barilius bendelisis* from a west flowing river Chalakudy in Kerala. Journal of the Bombay Natural History Society 95: 520-521.
129. Raju Thomas, K., C.R. Biju, and C.R. Ajithkumar. 1999. Additions to the fish fauna of Pambar River, Kerala. Journal of the Bombay Natural History Society 96: 332-334.
130. Raju Thomas, K., C.R. Biju, and C.R. Ajithkumar. 1999. Distribution of *Pangio goaensis* (Tilak) Cypriniformes: Cobitidae, in Manimala river, southern Kerala. Journal of the Bombay Natural History Society 96: 479.
131. Raju Thomas, K., C.R. Biju, and C.R. Ajithkumar. 1999. Extension of range of *Esomus thermoicos* (Pisces: Cyprinidae: Rasborinae) to Kerala. Journal of the Bombay Natural History Society 96: 163.
132. Raju Thomas, K., C.R. Biju, and C.R. Ajithkumar. 2000. Fish fauna of Idukki and Neyyar Wildlife Sanctuaries, southern Kerala, India. Journal of the Bombay Natural History Society 97: 443-445.
133. Raju Thomas, K., M.J. George, and C.R. Biju. 2002. Freshwater fishes of southern Kerala with notes on the distribution of endemic and endangered species. Journal of the Bombay Natural History Society 99: 47-53.
134. Ranjit Daniels, R.J. 2001. Endemic fishes of the Western Ghats and the Satpura hypothesis. Current Science 81: 240-244.
135. Rao, B.V.S. 1971. *Diodon holocanthus* from India. Journal of the Bombay Natural History Society 71: 390-394.
136. Rao, R.J. 2001. Biological resources of the Ganga River, India. Hydrobiologia 458: 159-168.
137. Rao, V.V. 1972. A new fish of the family Gobiidae from Godavari Estuary. Journal of the Bombay Natural History Society 69: 130-133.
138. Rao, V.V. 1974. Blenniid fishes from Godavari Estuary. Journal of the Bombay Natural History Society 71: 480-487.
139. Rao, D.M., and K.S. Rao. 1986. *Nemipterus peronii* (Valenciennes 1830) (Pisces: Nemipteridae) - a new record from Indian waters. Journal of the Bombay Natural History Society 83: 236-241.
140. Reddy, N.A.V.P., and C.U. Devi. 1982. Extension of range of *Enneapterygius obtusirostre*, with notes on its ecology. Journal of the Bombay Natural History Society 79: 696-698.
141. Reddy, N.A.V.P., and C.U. Devi. 1982. On a freak of *Istiblennius striatomaculatus*. Journal of the Bombay Natural History Society 79: 698-699.
142. Rema Devi, K., K.G. Emiliyamma, and R.S. Lalmohan, R.S. 2000. Range extension of *Pangio goaensis* (Cypriniformes: Cobitidae) to the Chaliyar drainage of Kerala. Journal of the Bombay Natural History Society 97: 150-152.
143. Rema Devi, K., and T.J. Indra. 2002. A note on *Mesonoemacheilus herrei* (Nalbant and Banarescu) (Cypriniformes: Balitoridae: Noemacheilinae). Journal of the Bombay Natural History Society 99: 333-336.
144. Rema Devi, K., and T.J. Indra. 2006. Occurrence of *Gagata itchkeea* (Siluriformes: Sisoridae) from Pennar River, Andhra Pradesh, India. Journal of the Bombay Natural History Society 103: 112-113.
145. Rema Devi, K., T.J. Indra, and S. Krishnan. 2002. On the distribution of *Oreonectes evezardi* and *O. keralensis* (Rita, Banarescu and Nalbant) (Pisces: Balitoridae). Journal of the Bombay Natural History Society 99: 127-129.
146. Rema Devi, K., T.J. Indra, and M.B. Raghunathan. 2007. Ichthyofauna of Indira Gandhi Wildlife Sanctuary, Tamil Nadu. Records of the Zoological Survey of India, Occasional Paper No. 277: 1-42.
147. Rema Devi, K., T.J. Indra, M.B. Raghunathan, and P.M. Raagam. 2005. A note on *Barilius* *bakeri* (Cyprinidae: Danioninae) from Karnataka with remarks on the status of *Opsarius malabaricus* (Jerdon). Journal of the Bombay Natural History Society 102: 123-124.
148. Rema Devi, K., and S. Krishnan. 2005. Observations on two catfish species from Bangalore District, Karnataka. Journal of the Bombay Natural History Society 102: 125.
149. Rema Devi, K., and A.G.K. Menon. 1994. *Horalabiosa palaniensis*, a new cyprinid fish from Palani Hills, Western Ghats, South India. Journal of the Bombay Natural History Society 91: 110-111.
150. Sahib, S.S. 2004. Fish diversity in the Kallada River (Kerala state). Journal of Advanced Zoology 25: 37-39.
151. Sarkar, S.K., B.D. Bhattacharya, A. Bhattacharya, M. Chatterjee, A. Alam, K.K. Satpathy, and M.P. Jonathan. 2008. Occurrence, distribution and possible sources of organochlorine pesticide residues in tropical coastal environment of India: an overview. Environment International 34: 1062-1071.
152. Sarkar, U.K., R.S. Negi, P.K. Deepak, W.S. Lakra, and S.K. Paul. 2008. Biological parameters of the endangered fish *Chitala chitala* (Osteoglossiformes: Notopteridae) from some Indian rivers. Fisheries Research 90: 170 -177.
153. Sarkar, U.K., A.K. Pathak, and W.S. Lakra. 2008. Conservation of freshwater fish resources of India: new approaches, assessment and challenges. Biodiversity and Conservation 17: 2495-2511.
154. Sekhar, K.C., N.S. Chary, C.T. Kamala, D.S.S. Raj, and A.S. Rao. 2004. Fractionation studies and bioaccumulation of sediment-bound heavy metals in Kolleru lake by edible fish. Environment International 29: 1001-1008.
155. Shaji, C.P., L.K. Arun, and P.S. Easa. 1996. *Garra surendranathanii* - a new cyprinid fish from the southern Western Ghats, India. Journal of the Bombay Natural History Society. 93: 572-575.
156. Shaji, C.P., and P.S. Easa. 1995. Extension of range of *Noemacheilus* (*Mesonoemacheilus*) *petrubanarescui* (Menon). Journal of the Bombay Natural History Society 92: 427-428.
157. Shaji, C.P., P.S. Easa, and S.C. Basha. 1995. Fresh water fish diversity in Aralam Wildlife Sanctuary, Kerala, South India. Journal of the Bombay Natural History Society 92: 360-363.
158. Singh, D., and R.C. Sharma. 1998. Biodiversity, ecological status and conservation priority of the fish of the River Alaknanada, a parent stream of the River Ganges (India). Aquatic Conservation: Marine and Freshwater Ecosystems 8: 761-772.
159. Singh, D.F., and G.M. Yazdani. 1992. *Osteobrama bhimensis*, a new cyprinid fish from Bhima river, Pune district, Maharashtra. Journal of the Bombay Natural History Society 89: 96-99.
160. Smakhtin V., M. Arunachalam, S. Behera, A. Chatterjee, S. Das, P. Guatam, G.D. Joshi, K.G. Sivaramakrishnan, and K.S. Unni. 2007. Developing procedures for assessment of ecological status of Indian river basins in the context of environmental water requirements. International Water Management Institute Research Report 114.
161. Sreekantha, M.D., S. Chandran, D.K. Mesta, G.R. Rao, K.V. Gururaja, and T.V. Ramachandra. 2007. Fish diversity in relation to landscape and vegetation in central Western Ghats, India. Current Science 92: 1592-1603.
162. Srinivasan R., and A. Sreenivasan. 1977. Fishery of the Cauvery River System, Tamilnadu, India. Proceedings of the Journal of Pacific Fishery Commission 17: 167-172.
163. Sritha, R.T., and K.C. Jayaram. 1990. On a new species of *Salmostoma* (Swainson) (Cyprinidae: Cultrinae) from Dhom Reservoir, Satara District, Maharashtra. Journal of the Bombay Natural History Society 87: 272-274.
164. Sujatha, K. 1988. Occurrence of *Anthias taeniatus* (Pisces: Serranidae) in Indian waters. Journal of the Bombay Natural History Society 86: 257-258.
165. Tehsin, R., V.S. Durve, and M. Kulshreshtha. 1988. Occurrence of a schizothoracine fish (snow trout) in a subterranean cave near Udaipur, Rajasthan. Journal of the Bombay Natural History Society 85: 211-212.
166. Thakre, V.Y., and S.S. Bapat. 1981. Maturation and spawning of *Rasbora daniconius* (Hamilton and Buchanan). Journal of the Bombay Natural History Society 78: 38-45.
167. Tilak, R., and A. Husain. 1990. Description of a new cyprinid, *Barilius dimorphicus* (Subfamily: Rasborinae) from Rajaji National Park, Uttar Pradesh. Journal of the Bombay Natural History Society 87: 102-105.
168. Tripathy, A.K., and R.K. Das. 1995. River water contaminated with paper mill effluent induces micronuclei in peripheral erythrocytes of fish. Current Science 68: 766-767.
169. University of Alberta: University of Alberta Ichthyology Collection
170. Uniyal, D.P., and A. Kumar. 2006. Fish diversity in selected streams of Chakrata and Shiwalik hills (District: Dehradun, Uttarachal), India. Records of the Zoological Survey of India, Occasional Paper No. 253: 1-120.
171. Vairavel, S.M., C.P. Shaji, and P.S. Essa. 1997. *Hypselobarbus kolus* - an addition to Kerala. Journal of the Bombay Natural History Society 95: 130.
172. Vass, K.K., M.K. Das, P.K. Srivastava, and S. Dey. 2009. Assessing the impact of climate change on inland fisheries in River Ganga and its plains in India. Aquatic Ecosystem Health and Management 12: 138-151.
173. Vass, K.K., H.S. Raina, and S. Sunder. 1978. On the breeding behavior of *Schizothorax niger* (Heckel) from Dal Lake. Journal of the Bombay Natural History Society 76: 179-184.
174. Vijaylaxmi, C., K. Vijaykumar, P. Zeba, and C. A. Kumar. 2008. Fish fauna of Bheema River, Gulbarga District, Karnataka. Journal of Ecophysiology and Occupational Health 8: 237-240.
175. Vishwanath, W., and W. Manojkumar. 1995. Fishes of the cyprinoid genus *Psilorhynchus* (McClelland) from Manipur, India, with description of a new species. Japanese Journal of Ichthyology 42: 249-253.
176. Wankhede, H. 2005. On a new species of the genus *Gangesia* (Gangesia), Woodland (1924) from fresh water fish *Mastacenbalus armatus* from Godavari River at Aurangabad, M.S. India, p. 103-107 *In* Biodiversity & Conservation (A. Kumar, ed.). A.P.H. Publishing Corporation, New Delhi, India.
177. Yadav, B.E. 2003. Ichthyofauna of northern part of Western Ghats. Records of the Zoological Surevey of India, Occasional Paper No. 215: 1-40.
178. Yazdani, G.M., and D.F. Singh. 1990. On the fish resources of Ujni wetland, Pune, Maharashtra. Journal of the Bombay Natural History Society 87: 157-161.
179. Zacharias, V.J., A.K. Bhardwaj, and P.C. Jacob. 1996. Fish fauna of Periyar Tiger Reserve. Journal of the Bombay Natural History Society 93: 39-43.
